# The Disease Process, Diagnosis and Treatment of Invasive Cervical Resorption: A Review

**DOI:** 10.3390/dj8030064

**Published:** 2020-07-01

**Authors:** Olivia Rotondi, PhiAnh Waldon, Sahng G. Kim

**Affiliations:** Columbia University College of Dental Medicine Division of Endodontics, Columbia University, New York, NY 10032, USA; oar2113@cumc.columbia.edu (O.R.); pw2477@cumc.columbia.edu (P.W.)

**Keywords:** invasive cervical resorption, calcium silicate-based cements

## Abstract

Invasive cervical resorption (ICR) is a localized, subepithelial, supra-osseous resorptive process of the tooth. Although there are several predisposing factors associated with ICR, its etiology and pathogenesis are poorly understood. The damage to the protective layer on the external root surface appears to allow for the attachment of clastic cells and initiate the resorptive process, which is confined by the inner protective pericanalar resorption-resistant sheet surrounding the root canal space. The use of cone-beam computed tomography (CBCT) is recommended for the diagnosis and assessment of a resorptive lesion. Based on the thorough evaluation of the size and location of the ICR lesion using CBCT, surgical or nonsurgical treatment can be chosen to address the source of the resorption. This review discusses the current status of knowledge regarding the biology of ICR lesions as well as their external or internal treatment using hydraulic calcium silicate-based materials. Future clinical outcome studies are necessary to evaluate the impact of hydraulic calcium silicate-based materials on the healing of ICR lesions.

## 1. Introduction

Invasive cervical resorption (ICR) is a pathologic resorptive process that initiates on the external surface of a tooth above alveolar bone crest and gradually replaces mineralized tooth structure with granulomatous fibro-vascular tissue or fibro-osseous tissue. It is a rare disease of which the prevalence ranges from 0.02% to 2.3% [1,2,3], but often leads to tooth loss due to its insidious and asymptomatic nature. The resorptive process of ICR is preceded by the loss of unmineralized precementum layers of the root and characterized by the presence of inflammation and odontoclastic activities below the epithelial attachment of gingiva. Its progression is limited in the extracanal area of the root and rarely invades the root canal space because of the presence of a protective layer surrounding the root canal space [4]. In order to capture the characteristics of this lesion, ICR has been described with various terms such as extracanal invasive resorption [5], supraosseous extracanal invasive resorption [6], external cervical resorption [7], peripheral inflammatory root resorption [8], subepithelial inflammatory resorption [9], and odontoclastoma [10].

Hydraulic calcium silicate-based materials have been recently suggested as a material to restore the resorptive defect of ICR. These materials have shown excellent biocompatibility with osteoinductive, cementoconductive and cementoinductive properties, which may promote the periodontal tissue regeneration or repair [11,12]. They are also known to provide an excellent sealing ability by inducing the formation of hydroxyapatite crystals and chemical bonds with the tooth structure [13]. The antibacterial effects of hydraulic calcium silicate-based materials during the setting reaction have been demonstrated in in vitro studies [14,15]. Despite the generally excellent material characteristics of hydraulic calcium silicate-based materials, not all seem suitable for the management of ICR. There is a growing body of literature concerning the repair of the lesions of ICR with selected hydraulic calcium silicate-based materials, which may allow for the more biocompatible restoration of the resorptive lesions that is conductive to periodontal tissue regeneration around ICR.

## 2. Literature Search and Scope of Review

Three electronic databases (PubMed, Embase, and Web of Science) were searched to identify relevant studies using appropriate MeSH terms including ‘invasive cervical resorption’, ‘extracanal invasive resorption’, ‘external cervical resorption’, ‘supraosseous extracanal invasive resorption’, ‘peripheral inflammatory root resorption’, ‘subepithelial inflammatory resorption’, ‘odontoclastoma’, ‘bioceramics’, ‘calcium silicate-based material’, ‘biodentine’, ‘mineral trioxide aggregate’, ‘endosequence root repair material’. The inclusion criteria were prospective and retrospective clinical studies, case reports, case series, histological studies, in vitro studies, and review articles that were pertinent to the mechanisms, predisposing factors, diagnosis and classification of ICR, and its management with calcium silicate-based materials. The exclusion criteria were studies based on surveys and studies not relevant to ICR. Of the 152 studies identified from the electronic database search, 59 studies are included for this review. A comprehensive narrative review of ICR was undertaken with relevant studies. The purpose of this study was to review the current knowledge concerning the disease process of ICR and internal and external management of ICR lesions with hydraulic calcium silicate-based materials.

## 3. Putative Mechanisms of the Disease Process of ICR

Studies on the etiology and pathogenesis of ICR are scarce due to its low prevalence. Two putative mechanisms may be proposed based on the histopathological findings from the ICR lesions [4,16,17]:

**Mechanism** **1.** *Microorganisms are not essential to the initiation of ICR but may be present as secondary invaders to sustain the resorptive process. Following the loss of protective unmineralized tissue (precementum), clastic cells circulating in the subepithelial connective tissue area attach the root surface. The microorganisms from the periodontal pocket that may invade the lesion can stimulate and sustain the inflammatory resorptive process;*

**Mechanism** **2.** *Microorganisms are essential to the initiation and perpetuation of ICR. Loss of protective unmineralized tissue (precementum) with concomitant inflammation caused by microorganisms in the periodontal tissue initiates the resorptive process at the cervical tooth structure. Microorganisms in the lesion contribute to the continuation of this resorptive process.*

The difference between the mechanisms lies in the role of microorganisms in the initiation of ICR. Recent histological observations seem to support the view that microorganisms are not essential to the initiation of the resorptive process and colonize the lesion at the later phase of the resorption based on the finding that bacteria are identified only at the outer layers of the lesions [16,17]. It is speculated that microorganisms may play a role in promoting the resorptive process by creating a hypoxic microenvironment in the lesion [16,17].

## 4. Predisposing Factors

The common contributor to ICR in both mechanisms is the loss of protective precementum layers. It is presumed that physical or chemical damage to precementum may increase the likelihood of ICR. Therefore, ICR can be considered a multifactorial disease, the prevalence of which is predisposed by factors associated with the damage to precementum. There are several predisposing factors reported in relation to the loss of the protective layers and ICR. Among these factors, trauma, orthodontic treatment, and periodontal treatment that could cause physical damage to precementum showed strong correlations with ICR [18,19,20,21,22,23]. Heithersay, in the analysis of predisposing factors from 222 patients with 257 ICR teeth, reported that orthodontic treatment (24.1%) was the most common factor, and trauma (15.1%) was the second most common [1]. A recent study by Jeng et al. revealed that dental or orofacial trauma (33.33%) were the most common factors associated with ICR, followed by periodontal treatment (26.98%) and orthodontic treatment (15.87%), based on the observation of 63 ICR teeth from 31 patients [19].

Internal bleaching, identified as a minor predisposing factor (3.9%) of ICR in the study by Heithersay, can cause chemical damage to the precementum layer [1]. It is likely that internal bleaching in the combination of other predisposing factors that cause physical damage to precementum increases the prevalence of ICR. Indeed, 7.4% of ICR teeth had a history of internal bleaching and trauma, and 13.6% showed a history of internal bleaching combined with trauma and/or orthodontic treatment [1]. It is hypothesized that bleaching agents such as 30% hydrogen peroxide penetrate into dentin and denature the organic components of dentin, cementum, and precementum, triggering a foreign body reaction around the cementoenamel junction (CEJ). This type of immune response allows for the recruitment of phagocytes and subsequent attachment of clastic cells, thus initiating the resorptive process via removing the chemically altered tooth structure, recognized as ‘foreign’ by the host immune system. It is noted that heating the bleaching agent to increase the efficiency of bleaching in the thermo-catalytic technique may augment the chemical alteration of tooth structure and promote the risk of ICR [24,25,26,27].

An anatomical defect between enamel and cementum at CEJ, which exposes underlying dentin, may also predispose teeth to ICR [28,29]. This developmental gap at CEJ is more susceptible to clastic activities, and the resorptive process may be initiated without other physical and chemical predisposing factors. The finding that no predisposing factors were identified in 16.4% of ICR teeth in the study by Heithersay [1] might suggest the potential contribution of this anatomical profile at CEJ to the development of ICR. It is presumed that internal bleaching increases the risk of ICR if this developmental defect exists at CEJ. Systemic sclerosis [30], hypercalciuria [31], feline herpes virus type 1 [32], varicella zoster virus [33], hepatitis B virus [34], and bisphosphonates [35] also have been reported to be associated with ICR.

A recent retrospective case-control study showed that the prevalence of ICR was 2.3% based on a 10-year observation of endodontic patients at the university clinic [3]. The disease prevalence of ICR is closely associated with predisposing factors that can cause the loss of the protective layers. Predisposing factors were identified in approximately 78% patients with ICR lesions in this study. Another recent study showed that predisposing factors were detected in 99% of ICR cases [36]. The maxillary anterior teeth were highly affected among all tooth types [3,18,36], implying that mechanical and chemical predisposing factors such as traumatic injuries and internal bleaching could be more frequently associated with this tooth type. No predilection for patients’ age and sex was identified in clinical studies [18,36].

## 5. Differences in the Pattern of ICR between Vital Teeth and Endodontically Treated Teeth

Why do ICR lesions rarely invade pulp space? It is speculated that there is a protective layer around the root canal space to inhibit the resorptive process, and unmineralized predentin has been thought to function as the protective layer. Mavridou et al., based on their histological analyses of ICR lesions, suggested that predentin and surrounding dentin could function as the protective layer and coined it as ‘pericanalar resorption-resistant sheet (PRRS)’ [17]. The resistance of PRRS to resorption may be attributed to its lower mineral content that inhibits the attachment of clastic cells and normal oxygen tension from blood supply in pulp tissue that attenuates clastic activities [17]. It is hypothesized that hypoxic microenvironments in the inflamed periodontal tissue at the initial phase of ICR promote osteoclastic activities, angiogenesis, and granulomatous tissue formation [37,38,39]. As the resorptive lesion approaches the pulp tissue at the later phase, PRRS, which provides normoxic microenvironments, prevents the lesion from entering the root canal space [17].

There are two notable differences reported in the pattern of ICR lesions between endodontically treated teeth and vital teeth.

1. Intensity of resorption
  More extensive resorption is observed in endodontically treated teeth than in vital teeth. This may be because, during endodontic therapy, part of PRRS is damaged mechanically or altered chemically [16]. Moreover, loss of pulp vitality in endodontically treated teeth may create hypoxic microenvironments conducive to continued osteoclastic activities [16,17];
2. Presence of osseous tissue
  Ingrowth of osseous tissue in the resorptive defect is more often observed in vital teeth than in endodontically treated teeth [16,17]. Hypoxic conditions induced by pulp tissue extirpation may contribute to the continued growth of granulomatous tissue formation by attenuating osteoblast growth and differentiation [40,41], and stimulating osteoclastic activities [37]. On the other hand, vital teeth maintain normoxic microenvironments around PRRS and allow for the osseous replacement of granulomatous tissue in the resorptive site during normal bone remodeling at the late phase of ICR [17]. Osseous tissue substituted for and integrated into part of PRRS may also function as the protective layer [17].

## 6. Diagnosis and Assessment of ICR

**Figure 1:**
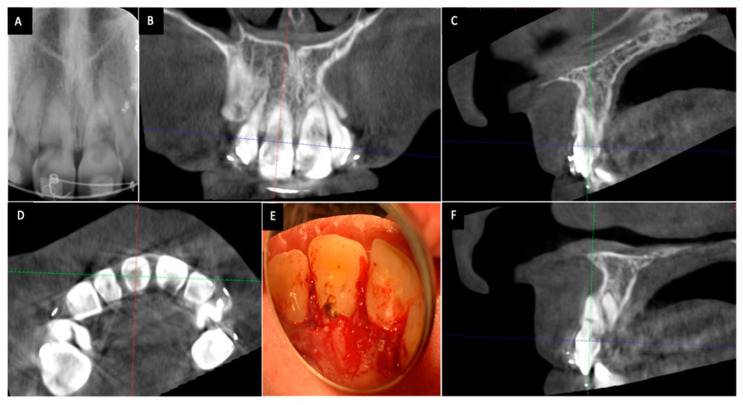
Cone-beam computed tomography (CBCT) shows a more accurate assessment of invasive cervical resorption (ICR) lesions than two-dimensional radiographs. The size and locations of ICR lesions can be determined with CBCT. (**A**) Periapical radiograph showing ICR lesions in maxillary right and left incisors; (**B**) The coronal view of CBCT scan image showing ICR lesions in maxillary right and left incisors;(**C**) The sagittal view of CBCT scan image (maxillary right incisor); (**D**) The axial view of CBCT scan image showing the ICR lesion in maxillary right and left incisors; (**E**) Clinical view of ICR lesions located on the palatal surface of the teeth; (**F**) The sagittal view of CBCT scan image showing the ICR lesion in maxillary left incisor.

A correct diagnosis and assessment of ICR is an essential prerequisite to its successful treatment. Identification and assessment of ICR lesions heavily rely on radiographic interpretation due to their presence in the subgingival areas. ICR lesions can spread axially, horizontally, and circumferentially as they penetrate into dentin [42]. The sizes and locations of ICR lesions may not be accurately assessed when only two-dimensional radiographs are used (Figure 1). A clinical study by Patel et al. compared the effectiveness of periapical radiographs with that of cone-beam computed tomography (CBCT) in the diagnosis and assessment of ICR [43]. Receiver operating characteristic curve analysis in this study revealed that periapical radiographs had limited performance in the assessment of the size, circumferential spread and locations of the lesions compared with CBCT [43]. Furthermore, periapical radiographs showed significantly lower sensitivity and specificity than CBCT for the detection of ICR lesions [43]. The superiority of CBCT over periapical radiographs for the diagnosis of resorptive defects has also been demonstrated in in vitro studies [44,45,46]. It is highly recommended that CBCT should be used to diagnose and assess the resorptive lesions prior to treatment as stated in American Association of Endodontists/American Association of Oral and Maxillofacial Radiology Joint Position Statement [47] and European Society of Endodontology Position Statement [48] concerning the use of CBCT in Endodontics.

## 7. Classification of ICR

The classification of ICR lesions has been first developed by Heithersay for the basis of clinical assessment and research purposes [4]. ICR lesions are classified into four categories based on the size and extension of resorptive defects into dentin: Class 1, a small resorptive lesion at the cervical area with superficial penetration into dentin; Class 2, a more invasive resorptive lesion toward the coronal pulp with little or no involvement of radicular dentin; Class 3, a deep cervical resorptive lesion with the extension into the coronal third of radicular dentin; Class 4, an extensive resorptive lesion beyond the coronal third of radicular dentin. Despite the usefulness of this classification, the lesions may not be correctly classified when two-dimensional radiographs were used. Indeed, an ex vivo study by Vaz de Souza et al. showed that examiners had correctly classified 39.7% of cases with periapical radiographs, while approximately 70% cases with CBCT [49]. Moreover, there was a significantly higher intra- and inter-examiner reproducibility associated with CBCT compared to periapical radiographs [49]. To overcome the difficulties in classifying and assessing ICR lesions, Patel et al. have recently devised a three-dimensional classification method using CBCT [50]. This new classification considers three parameters, including the height of ICR lesions, the circumference of the lesions, and proximity of the lesions to root canals [50]. The height of the lesions is scored as supracrestal (1), subcrestal extending into the coronal third of radicular dentin (2), extending into the middle third of radicular dentin (3), and extending into the apical third of radicular dentin (4). The circumference of the lesions is rated as smaller than 90° (**A**), between 90° and 180° (**B**), between 180° and 270° (**C**), and greater than 270° (**D**). The proximity of the lesions to the root canal is graded as within dentin (d) and pulp involvement (p). The grading value from each parameter is combined to describe the size and extension of an ICR lesion three-dimensionally. This classification system seems to provide a more accurate assessment of the preoperative condition of ICR than the Heithersay classification and may help clinicians to have better treatment planning and management of the lesions.

## 8. Strategies to Manage ICR Lesions

The management of ICR lesions has focused on removing clastic cells to arrest the resorptive process and proper restoration to maintain a tooth’s structural integrity. Recently, how to reconstitute the periodontal apparatus around the resorption site also has become a new research focus. There are two main strategies proposed based on the method to arrest clastic activities: an external approach and internal approach. The external approach is comprised of surgical and nonsurgical treatment. When the lesion is accessed externally, surgical treatment, including flap surgery, may be necessary if the lesion is located and extended far below the gingival margin. However, external nonsurgical treatment can be performed if the lesion is located at the gingival margin or not extended below the alveolar bone crest. The internal approach is used to access the lesions non-surgically through the endodontic access cavity regardless of the locations of the lesions.

### 8.1. External Approach

Heithersay first described an external approach, including the topical application of trichloroacetic acid (TCA) to resorptive lesions, curettage of the lesions, endodontic treatment if indicated, and restoration with glass ionomer (GI) [4,51]. This nonsurgical external treatment can be performed on teeth with ICR lesions located above the coronal third of the root, but a surgical technique including periodontal flap surgery is usually performed if the lesions extend below the gingival margin. The surgical external approaches have been more commonly reported than nonsurgical external approaches perhaps due to the extensive size of the lesions at the time of detection. The surgical techniques reported in studies include removal of the resorptive lesions using TCA and/or mechanical debridement and restoration with hydraulic calcium silicate-based materials or glass ionomer cement [51,52,53].

Due to recent advances in materials and instruments, the techniques for the management of ICR lesions should be revisited with the objective of promoting healing processes and regeneration. TCA has been widely used in the management of ICR lesions. It is known to induce coagulation necrosis of the lesions [54], thereby facilitating the removal of the resorptive tissue [51]. However, it is very caustic and induces inflammation in surrounding periodontal tissue. In addition, it can remove hydroxyapatites from the dentin surface to a great extent and will compromise the bonding strength of glass ionomer (GI) [55]. The use of ultrasonic instruments under a surgical microscope may eliminate the need for TCA because it can access small internal resorptive areas without removing a significant amount of tooth structure and safely debride the lesions without irritating periodontal tissue. The use of burs should be minimized to preserve an intact tooth structure around the resorptive lesion.

GI or resin-modified glass ionomer (RMGI) has been advocated as a restorative material for the resorptive defect due to its desirable material properties such as biocompatibility, hydrophilicity, fluoride release, and chemical adhesion to dentin [56,57,58]. The subgingival placement of GI or RMGI also allows for gingival attachment [59,60], but does not induce the regeneration of cementum or bone around the materials. Hydraulic calcium silicate-based materials such as Biodentine (Septodont, Saint-Maur-des-Fossés, France) and Endosequence Root Repair Material (Brasseler, Savannah, GA, USA) have been recently adopted to restore the resorptive cavities. The main advantage of these materials over GI or RMGI is the ability to regenerate cementum, periodontal ligament, and bone [11,12,13]. If the resorptive defect is subgingival or subcrestal and accompanies periodontal tissue destruction, it is preferable to use hydraulic calcium silicate-based materials. Several case reports have been published to support the use of hydraulic calcium silicate-based materials for the restoration of ICR defects [52,53,61,62,63,64,65].

The selection of hydraulic calcium silicate-based materials for restoring ICR defects should be based on their setting time and mechanical properties such as microhardness, bond strength, washout resistance, and compressive strength because ICR defects are located around the mechanically challenging cervical area of a tooth. It is more advantageous to select the materials such as Biodentine or mineral trioxide aggregate (MTA) (ProRoot MTA, Dentsply, York, PA, USA) with a short setting time [66,67] because it allows for better retention with minimal material loss. Other fast setting materials such as Endosequence Root Repair Material-Fast Set Putty may also be considered. Concerning the mechanical properties, Biodentine seems to be a good potential candidate for the restoration of ICR defects [68]. It showed higher surface microhardness compared with GI or RMGI [69]. The push-out bond strength of Biodentine was found to be higher than MTA [70] and was not significantly affected by blood contamination [70] or endodontic irrigants such as sodium hypochlorite and chlorhexidine [71]. It also demonstrated higher washout resistance and compressive strength than MTA [66]. Hydroxyapatite crystal growth at the interface between Biodentine and dentin was confirmed by scanning electron microscopy, suggesting that the seal could improve over time [72]. In addition, it showed significantly less discoloration than MTA, preventing an aesthetic complication in anterior teeth [73]. The chemical composition of ProRoot MTA, Biodentine, and Endosequence Root Repair Material was presented in Table 1 [74,75].

Orthodontic extrusion can be an alternative to accessing the lesions without surgical interventions [55,76,77]. Intentional replantation has been recently proposed to manage the lesions that are difficult to access [77].

### 8.2. Internal Approach

It may sound unreasonable to treat ICR lesions internally because the source of resorption (clastic cells and blood supply) originates from and exists on the external surface of teeth, but nonsurgical internal methods to address the lesions by eliminating the source also have been proposed.

Salzano and Tirone successfully treated four cases with Heithersay Class 4 ICR lesions in their case series by using a nonsurgical internal approach [78]. The resorptive granulomatous tissue was mechanically removed through access cavities and root canals and was repaired with MTA or Biodentine following root canal treatment [78]. The survival of the four treated teeth was confirmed to have no relapse of resorption and normal periradicular bone healing at a follow-up varying from four months to 18 months [78]. Similar case reports and case series were reported to have promising outcomes at follow-ups longer than two years [79,80,81]. However, this approach has major shortcomings, including difficulty in removing all resorptive tissue through limited internal access and excessive removal of the tooth structure that may make treated teeth more susceptible to fracture.

Shemesh et al. showed the most conservative method to address Heithersay Class 4 ICR lesions in four maxillary anterior teeth [82]. They used ultrasonically activated sodium hypochlorite irrigation and four-week calcium hydroxide medication to dissolve and necrotize the resorptive tissue without mechanically removing the lesions. The root canals and resorptive defects were filled with gutta percha and sealer only without calcium silicate-based materials [82]. The teeth survived without radiographic progression of resorption at a three-year or five-year follow-up [82].

Another internal method using nonsurgical root canal treatment and obturation with a type of calcium silicate-based cements (calcium-enriched mixture cement) was suggested by Asgary and Nosrat in their case report [83]. A mandibular lateral incisor with a Heithersay Class 4 ICR lesion was endodontically treated in a single visit using chemomechanical instrumentation and filling with the calcium-enriched mixture cement [83]. Normal periapical and periradicular healing with arrested resorption was observed at a two-year follow-up [83]. It was suggested that calcium silicate-based cement placed in the root canal could deactivate clastic cells and arrest the resorptive process by releasing calcium hydroxide. It is presumed that calcium hydroxide used for four weeks prior to root canal filling in the case series by Shemesh et al. [82] may have a therapeutic effect on ICR lesions similar to that of the calcium silicate-based cement in this case report. A case report by Patni et al. used two-week calcium hydroxide medication followed by obturation with Biodentine for the treatment of maxillary lateral incisor with a Class 4 lesion [84]. The tooth was found to be asymptomatic and have periapical healing at a five-year follow-up [84].

A most recent case series suggested that teeth with ICR lesions and normal pulp could be treated by using vital pulp therapy with calcium-enriched mixture cement [85]. Five mandibular molars and one maxillary molar underwent partial pulp excavation to the level of ICR lesions followed by the application of calcium-enriched mixture cement in order to exert the therapeutic effect of the cement near the resorptive lesions [85]. All six teeth treated using vital pulp therapy survived without signs of new resorption at a follow-up period ranging from 12 to 36 months [85]. Alkalinization of the lesions by the sustained release of calcium hydroxide from the cement was thought to arrest clastic activities and promote the healing process [86].

Various internal approaches were proposed to manage ICR lesions [62,78,79,80,81,82,83,84,85]. In general, more extensive resorptive lesions have been managed with internal approaches [78,82,83] because external surgical or nonsurgical approaches require the complete excavation of lesions through the tooth structure that may compromise the long-term survival of treated teeth.

## 9. Prognosis

There is limited information regarding the outcomes of treatment for ICR lesions due to the scarcity of clinical outcome studies. A majority of studies are case reports and case series [8,20,21,52,53,55,62,63,64,65,78,79,80,81,82,83,84,85], and it may be difficult to predict the prognosis of treated teeth based on those studies because only successful cases tend to be reported. Furthermore, due to the disease rarity, clinical studies may seem difficult to perform.

A clinical study by Heithersay reported the outcomes of the external management of ICR lesions according to his classification of the lesions [51]. The external technique included the use of 90% TCA, curettage, root canal treatment when indicated, and GI restoration for resorptive defects. The outcome was assessed using the success criteria defined as periapical and periradicular healing with no signs of resorption. Based on the analysis of 101 affected teeth in 94 patients with at least three year follow-up period, 100% success was observed for teeth with class 1 and 2 lesions [51]. The success rate was 78% for class 3 lesions, and 2% for class 4 lesions [51]. The reported success rates suggest that the external approach is not advisable for teeth with class 4 lesions. The internal approach using hydraulic calcium silicate-based materials may be indicated for these extensive resorptive defects, although there are only a few successful case reports or case series available [62,78,79,80,81,82,83,84,85].

## 10. Concluding Remarks

ICR is an aggressive, subepithelial, supra-osseous resorptive process that gradually replaces the unprotective mineralized tooth structure with fibro-vascular tissue and/or fibrous-osseous tissue. The mechanism for ICR resorption is poorly understood, but various dental procedures that cause physical or chemical damages to unmineralized protective layers on the external surface of teeth seem to contribute to the initiation of resorption. Histopathologic analyses revealed the differences in the pattern of ICR between endodontically treated teeth and vital teeth. Due to the presence of PRRS and normoxic microenvironments, less invasive resorption with more fibrous osseous tissue is observed in vital teeth than in endodontically treated teeth. For the diagnosis and assessment of ICR lesions, CBCT is highly recommended because two-dimensional radiographs may underestimate the size of lesions and cannot correctly locate the lesions three-dimensionally. The removal of clastic cells in the resorptive lesions, followed by the restoration of the defect, is the main treatment strategy. Two main approaches have been proposed to address the clastic activities. External approaches have been more commonly used, since they are directed at the complete removal of the source of resorption and better sealing and restoration of the defects with bioactive materials such as hydraulic calcium silicate-based materials. However, the low success rates reported for the management of extensive ICR lesions deter the implementation of the external method and likely warrant a different approach. Internal approaches have been proposed as a new alternative, mainly for the management of the extensive lesions. The internal use of hydraulic calcium silicate-based materials may be suggested to reverse the resorptive process, as reported in case reports and case series. Clinical outcome studies are required to confirm the therapeutic effect of hydraulic calcium silicate-based materials on ICR lesions.

## Figures and Tables

**Table 1:** The chemical composition of ProRoot mineral trioxide aggregate (MTA), Biodentine, and Endosequence Root Repair Material.

Materials	Chemical Composition
ProRootMTA [74]	Tricalcium silicate, Dicalcium silicate, Tricalcium aluminate, Tetracalciumaluminoferrite, Calcium sulfate, Bismuth oxide, Calcium oxide, Silicon oxide, Aluminum oxide
Biodentine [74]	Tricalcium silicate, Dicalcium silicate, Calcium Carbonate, zirconium oxide, Iron oxide
Endosequence Root Repair Material [75]	Calcium silicate, Calcium phosphate monobasic, Zirconium oxide, Tantalum Oxide, filler and thickening agents
